# The Lon Protease Is Essential for Full Virulence in *Pseudomonas aeruginosa*

**DOI:** 10.1371/journal.pone.0049123

**Published:** 2012-11-07

**Authors:** Elena B. M. Breidenstein, Laure Janot, Janine Strehmel, Lucia Fernandez, Patrick K. Taylor, Irena Kukavica-Ibrulj, Shaan L. Gellatly, Roger C. Levesque, Joerg Overhage, Robert E. W. Hancock

**Affiliations:** 1 Centre for Microbial Diseases & Immunity Research, University of British Columbia, Vancouver, British Columbia, Canada; 2 Karlsruhe Institute of Technology (KIT), Institute of Functional Interfaces, Karlsruhe, Germany; 3 Institut de Biologie Intégrative et des Systèmes (IBIS), Université Laval, Québec City, Québec, Canada; Vrije Universiteit Brussel, Belgium

## Abstract

*Pseudomonas aeruginosa* PAO1 *lon* mutants are supersusceptible to ciprofloxacin, and exhibit a defect in cell division and in virulence-related properties, such as swarming, twitching and biofilm formation, despite the fact that the Lon protease is not a traditional regulator. Here we set out to investigate the influence of a *lon* mutation in a series of infection models. It was demonstrated that the *lon* mutant had a defect in cytotoxicity towards epithelial cells, was less virulent in an amoeba model as well as a mouse acute lung infection model, and impacted on *in vivo* survival in a rat model of chronic infection. Using qRT-PCR it was demonstrated that the *lon* mutation led to a down-regulation of Type III secretion genes. The Lon protease also influenced motility and biofilm formation in a mucin-rich environment. Thus alterations in several virulence-related processes *in vitro* in a *lon* mutant were reflected by defective virulence *in vivo*.

## Introduction

*Pseudomonas aeruginosa* is a versatile Gram-negative bacterium that is found in many natural environments (including soils and marshes) but also causes infections of animals and plants [1]. *P. aeruginosa* is a major opportunistic human pathogen, being the third most common cause of nosocomial infections. It can cause pneumonia, urinary-tract infections and bacteremia, as well as morbidity and mortality in cystic fibrosis (CF) patients due to chronic infections that eventually lead to lung damage and respiratory failure. *Pseudomonas* is resistant to multiple antibiotics and has consequently joined the ranks of “Superbugs”. Infections are difficult to eradicate due to its high intrinsic resistance, together with its ability to develop resistance to common antibiotics through adaptation and mutation [2].

Furthermore, *Pseudomonas* can form biofilms in the lung [3]. This social behaviour enables the organism to resist clearance by the immune system, since organisms in biofilms are not accessible to phagocytes, and in addition bacteria growing in a biofilm are substantially more resistant to antimicrobial agents [4], [5], [6]. In chronically infected patients, where *P. aeruginosa* is thought to grow as biofilms, the bacteria are highly adaptively resistant to antimicrobial treatment, making eradication very difficult.

Intracellular proteases have been shown to play a decisive role in the coordination of diverse cellular processes in bacteria, including *P. aeruginosa* [7], [8]. One such protein is the ATP-dependent Lon protease, which belongs to the AAA+ (ATPases associated with a variety of cellular activities) family. To date the structure and function of Lon has been best described in *Escherichia coli* [8]. In this microorganism, Lon together with ClpP is responsible for 70–80% of energy-dependent protein degradation [9]. Thus, Lon recognizes and binds to misfolded and degraded proteins present in the cytoplasm and, subsequently, peptide bond cleavage of the misfolded proteins occurs in the proteolytic chamber of the protease. The Lon protease of *P. aeruginosa* has 84% similarity to its *E. coli* homolog (www.pseudomonas.com); therefore, it is similarly expected to play a major role in protein quality control in this microorganism. Lon is a cytoplasmic serine protease of 87 kDa that contains a Ser_679_-Lys_722_ dyad, which is responsible for the proteolytic activity [10]. It is well known that the Lon proteases of Gram-negative bacteria associate into hexameric rings. Also well established is the fact that Lon proteases are divided into two subfamilies in the bacterial kingdom depending on the presence of an N-terminal domain. Thus, LonA members exhibit three domains: N-terminal domain for substrate recognition, ATP-binding domain and a C-terminal domain for proteolytic cleavage. In contrast, LonB members lack the N-terminal domain and possess instead a membrane anchor. Examples of LonA members are those of *E. coli* and *P. aeruginosa*, whereas *Archaeglobus fulgidus* has a LonB-type protease [11]. Attempts to determine the full crystal structure of the Lon protease in *E. coli* have failed so far, although structures corresponding to certain domains of this protease are available [10], [12], [13]. This has enabled studies that attempt to establish structure-function relations of the Lon protease. In the case of *P. aeruginosa*, no crystal structure is available yet; however, it is predicted to exhibit structure similarity to *E. coli* Lon. The Lon protease has been shown to play a role in virulence *in vivo* in several pathogenic bacteria. For instance, studies carried out in *Salmonella* and *Campylobacter* demonstrated that the lack of Lon leads to a less severe infection in various animal models, highlighting the importance of Lon in the virulence of these pathogens [14], [15], [16]. In *P. aeruginosa*, the involvement of the Lon protease in antibiotic resistance and virulence-related properties has already been described. Indeed, mutants in the ATP-dependent Lon protease of *P. aeruginosa* exhibited ciprofloxacin supersusceptibility, filamentation, deficiencies in swarming and twitching motilities as well as biofilm formation [17], [18], [19]. Furthermore, this protease regulates quorum-sensing, and a *lon* mutant exhibited increased hemolytic activity [20]. However, no study to date has demonstrated the importance of Lon for the virulence of *P. aeruginosa in vivo*.

In this study, the importance of the Lon protease in the virulence of *P. aeruginosa* was investigated using *in vitro* cell and *in vivo* infection models. Overall, our results demonstrated that Lon does indeed contribute to pathogenesis in *Pseudomonas* infections and provided mechanistic insight into its involvement in virulence.

## Materials and Methods

### Bacterial Strains and Growth Conditions

The strains used in this study are shown in Table 1, including *lon* transposon mutants and the complemented counterpart [21], [22]. Of note, most experiments described here were performed using a *P. aeruginosa* PAO1 background, unless stated otherwise. Growth was routinely performed in Luria-Bertani broth (LB) unless otherwise indicated.

**Table 1:** *P. aeruginosa* strains and plasmids used in this study.

Strain or plasmid	Description and characteristics^a^	Reference
**Strains**		
***P. aeruginosa***		
H103 (WT)	Wild-type *P. aeruginosa* PAO1; strain H103	lab collection
PAO1 *lon* mutant	PA01 *lon::*mini-Tn5-*luxCDABE*; mutant 74_D9, Tet^r^	[21]
PA14 (WT)	Wild-type *P. aeruginosa* PA14	lab collection
PA14 *lon* mutant	PA14 transposon insertion mutant, ID29158, Gm^r^	[22]
*lon*::p*lon^+^*	Complemented mutant PAO1 *lon*::mini-Tn5-*luxCDABE*(pBBR1MCS4::*lon* ^+^), Cb^r^	[42]
***E. coli***		
TOP10	F^–^ *mcrA* Δ(*mrr*-*hsdRMS*-*mcrBC*) φ80*lacZ*M15 Δ*lacX74 recA1 ara*Δ*139*Δ(*ara*-*leu*)*7697 galU galK rpsL* (Str^r^) *endA1 nupG*	Invitrogen
DH5α	F^–^φ80*lacZ*ΔM15 Δ(*lacZYA-argF)U169 deoR recA1* *endA1 hsdR17*(r_K_^–^ m_K_^+^) *supE44*λ^–^ *thi-1 gyrA96 relA*	Invitrogen
**Plasmids**		
pCR-Blunt II-TOPO	PCR cloning vector; Kan^r^	Invitrogen
pBBR1MCS4	Low copy number broad-host-range cloning vector; Amp^r^	[43]
pBBR1MCS4:*lon*+	Cloning vector harbouring the *lon* amplicon; Amp^r^	[42]

a Antibiotic resistance phenotypes: Amp^r^, ampicillin resistance for *E. coli*; Cb^r^, carbenicillin resistance for *P. aeruginosa*; Kan^r^, kanamycin resistance; Tet^r^, tetracycline resistance Gm^r^, gentamicin resistance.

### Cytotoxicity

The lactate dehydrogenase (LDH) release assay was used to quantify bacterial induced killing of the human bronchial epithelial cell line 16HBE14o^−^ (HBE), kindly provided by Dr. D. Gruenert (University of California, USA) [23]. HBE cells were grown at 37°C with 5% CO_2_ in minimum essential medium (MEM) (Invitrogen) supplemented with 10% fetal bovine serum (FBS) and 2 mM L-glutamine (Invitrogen) until 90% confluency was reached. The day of the experiment, the HBE cells were washed and rested for 1 hour at 37°C in MEM containing 1% FBS and 2 mM L-glutamine. Wild type and the *lon* mutant were grown in LB to mid-logarithmic phase, washed with phosphate buffered saline (PBS) and resuspended in MEM. This was followed by adding bacteria at a multiplicity of infection (MOI) of 50 bacteria per HBE cell and incubation at 37°C in the presence of 5% CO_2_. At 10 and 18 hours post-infection, the supernatant was collected, centrifuged to pellet the bacteria and supernatant stored at 4°C. LDH levels in the bacteria-free supernatant were determined using a cytotoxicity detection kit (Roche) as per the manufacturer’s instructions. The absorbance at 490 nm was measured after 30 minutes, and HBE cells untreated and treated with 2% Triton X-100 served as the negative and positive controls, respectively. Percent cytotoxicity was calculated using the formula: % cytotoxicity  = 100 × [(E – N)/(P – N)], where E, N, and P denote experimental absorbance, vehicle treatment absorbance, and 2% Triton X-100 absorbance, respectively.

### Adhesion Assay

HBE cells were grown as described above and incubated (MOI = 50) with mid-logarithmic phase bacterial cultures. Samples were incubated at 37°C in a 5% CO_2_ atmosphere for 3 hours, at which point the monolayers were washed with PBS, and the cells were fixed with 4% formaldehyde in PBS for microscopic analysis. For morphological determination, the microscope slides were stained with Diff-Quick (Siemens) and attachment to HBE cells was determined. The co-cultures were visualised on a Nikon Eclipse TE2000-5 with a CoolSnap ES camera and Image Pro Plus software.

### Amoeba Virulence Model

*Dictyostelium discoideum* A×2 was routinely grown in HL5 medium in cell culture flasks at 22.5°C as described previously [24]. The assay was performed according to the method described previously [25]. Briefly, 50 µl of overnight cultures of *P. aeruginosa* wild type, the *lon* mutant or the complemented strain grown in LB medium were mixed with 200 µl PBS buffer and plated on M9 agar plates (6 g Na_2_HPO_4_, 3 g KH_2_PO_4_, 0.5 g NaCl, 1 g NH_4_Cl, 1 mM MgSO_4_, 0.1 mM CaCl_2_, 0.4% glucose, and 15 g agar per liter). Plates were dried in a laminar flow hood for 1 hour at 23°C to obtain a dry and even bacterial lawn. Amoebae, grown for 2–4 days in HL5 medium, were harvested by centrifugation at 1,600×g for 10 minutes, washed and resuspended in PBS buffer. Cells were adjusted to 8×10^6^ cells per ml and kept on ice. This stock solution was used to prepare droplets of 5 µl containing different numbers of amoebae from 20,000 down to 20 amoebae, which were subsequently spotted onto the bacterial lawn. The plates were incubated at 22.5°C for 5 days. The highest dilution at which growth of the amoebae formed a visible plaque was recorded. Experiments were carried out at least in triplicate with two technical repeats each time.

### Competitive Index (CI) Determination

For *in vitro* CI assays, overnight cultures of the *lon* mutant tagged with the mini Tn5 tet^R^ marker were mixed (1∶1) with the PAO1 wild type or the complemented *lon* strain. Bacterial mixtures were incubated, under shaking conditions, for 8 hours at 37°C. Bacterial mixtures were then plated for CFU counts on plates containing LB with the addition of tetracycline for the *lon* mutant, carbenicillin for the complemented strain or no antibiotic for total counts. The latter were used to calculate the CFUs corresponding to the wild type by substracting the total CFU counts from the number of CFUs that grew on the antibiotic-containing plates. CFU counts were determined after 18 hours incubation at 37°C.

The rat chronic lung infection model in which the bacteria were enmeshed in agar beads [26] was used to measure the CI *in vivo*. This study was performed in strict accordance with the recommendations in the Guide for the Care and Use of Laboratory Animals at Université Laval. The protocol was approved by the Committee on the Ethics of Animal Experiments of Université Laval (Permit Number: 2011194-1). All surgery was performed under anesthesia as recommended, and every effort was made to minimize suffering. Cultures of the wild type, *lon* mutant and complemented strain were grown in Tryptic soy broth (BD) overnight at 37°C and diluted to a final concentration of 1×10^10^ cfu/ml. A 1∶1 mixture of either the PAO1 wild type or the complemented strain with the *lon* mutant was added to agarose in PBS at 48°C. The agarose-bacterial mixture was added to heavy mineral oil and stirred on a magnetic stirrer in a water bath. The mixture was then cooled to 0°C on ice, at which point the bacteria-containing agarose beads were washed with sodium deoxycholate and PBS, incubated on ice, and the residual PBS then removed. Male Sprague-Dawley rats were anaesthetized using isofluorane and intubated with 120 µl of the agarose bead suspension (containing 10^5^ bacteria). The animals were sacrificed at 7 days post-infection and the lungs were removed, homogenized and plated on antibiotic-containing media as mentioned above to determine CFU counts. The CFU counts represented the total number of bacterial cells from each strain present in the lungs of the rats. The CI was defined as the CFU output ratio of mutant compared to the wild type, divided by the CFU input ratio of mutant compared to the wild type [27]. The Mann Whitney test was used to determine statistical significance between the mutant and the wild type.

### Mouse Model of Acute Lung Infection

All mouse experiments were conducted in accordance with the Animal Care Ethics Approval and Guidelines of the University of British Columbia (Protocol number: A10-0261). The acute lung infection model was adapted with modifications from the protocol of Balloy et al. [28]. To date, this model has been well established for the more virulent PA14 strain. Therefore, strains with this background were used in these experiments instead of the PAO1-based strains. CD-1 female mice (supplied by Harlan Laboratories) were maintained under specific pathogen-free conditions. Mice were 6 weeks of age and weight matched in all experiments. CD-1 mice were anaesthetized via inhalation of aerosolized isoflurane (2 to 5%) mixed with oxygen and infected by intranasal administration with a fresh culture of *P. aeruginosa* resuspended at 10^6^ CFU/20 µl PBS for the PA14 WT and the *lon* mutant. At 4 hours post-infection, mice were euthanized by lethal pentobarbital injection (120 mg/kg, IP). The bronchoalveolar lavage (BAL) was collected by cannulating the trachea and washing the lung 1 time with 600 µl of saline solution. Bacterial numbers in the BAL were measured by serial dilution and plating on LB-agar plates.

### Reverse Transcription Reaction

Total RNA from the wild type and the *lon* mutant was prepared from cultures grown under various conditions as follows: (i) for the liquid culture, strains were grown in LB to mid-logarithmic phase (OD 0.5–0.6), and then harvested for RNA extraction; (ii) for swarming conditions, cells were grown in BM2 to mid-logarithmic phase and then inoculated onto swarming plates as described below. Swarming plates were incubated overnight at 37°C. The following day, cells were collected from the edge of the swarming colonies and used for RNA isolation. For each sample, 1 µg of DNase treated RNA was mixed with random primers (Invitrogen) and incubated at 70°C for 10 minutes, followed by incubation at 25°C for 10 minutes. RT buffer (1×), 10 mM DTT, 10 mM dNTPs, 30 U SUPERase-IN and 1500 U Superscript II reverse transcriptase were added to the RNA. Samples were then incubated at 37°C for 1 hour, 42°C for 3 hours, and finally 72°C for 10 minutes. Residual RNA was removed from the cDNA by incubating with 1 N NaOH for 15 minutes at 65°C. NaOH was then neutralized with 1 N HCl. The prepared samples were used for real-time PCR.

### Real-time PCR (qPCR)

The ABI Prism 7000 sequence detection system and the SYBR green dye (Applied Biosystems) were used to perform real-time PCR to validate the microarray data and analyze transcriptional changes under stress conditions. Primers were designed using ABI Primer Express v2.0 and the sequence of the gene of interest. cDNA was prepared using a reverse transcription reaction, diluted (1∶100) in nuclease-free water (Ambion), and mixed (2.5 µl) with 1 × SYBR Master Mix (Applied Biosystems) and 10 µM of each primer. Samples were loaded, in triplicate, in 96 well plates, analyzed with the ABI Prism 7000 sequence detection system and the average fold change (FC) in expression were calculated using the following formula where CT denotes the gene expression values:

ΔCTtest  =  CT test – CT housekeeping gene; ΔCTcontrol  =  CT control – CT housekeeping gene; ΔΔCT  =  ΔCTtest - ΔCTcontrol; FC = 2^−ΔΔCT^. The genes used for normalization were *anr* (PA1544) for swarming conditions [29] and *rpsL* (PA4268) or PA2018 (efflux transporter) for liquid cultures. In all cases, normalization was done by using a gene that showed no dysregulation under the relevant conditions.

### Swarming Motility

Swarming motility was assessed by inoculating a bacterial culture (1 µl) onto a swarming plate (modified BM2 medium +0.5% agar). In the modified BM2 medium, ammonium sulfate was substituted with 0.5% casamino acids. The swarming behaviour (dendritic colonial appearance) of mutant strains was compared to that of the wild-type strain after 20 hours of incubation at 37°C [30].

### Surfing Motility

To mimic the lung environment, where the epithelium is overlayed by a viscous mucous layer, mucin (Sigma-Aldrich), the major glycopeptide from the lung mucous layer was added at various concentrations (0.1, 0.5 and 1%) to 0.4% BM2 agar motility plates (with and without ciprofloxacin). Mid-logarithmic bacterial cultures (1 µl) were inoculated onto the agar plates. Motility and antibiotic resistance were assayed after 20 hours of incubation.

### Biofilm Formation

The abiotic solid surface assay was used to measure rapid bacterial attachment [31], [32]. Overnight PAO1 cultures were diluted (1∶100) in LB broth and normalized for cell density. Cultures were transferred to 96-well polystyrene microtiter plates, which were incubated for 1 hour (rapid attachment) or 24 hours (mature biofilm formation) under static conditions at 37°C. Crystal violet (Sigma-Aldrich) was used to stain the attached cells, followed by the addition of ethanol to extract the crystal violet, and the absorbance (595 nm) was measured on a Microtiter plate reader (Bio-Tek Instruments).

## Results

### Reduced Virulence of the *lon* Mutant in Cellular Models

**Figure 1:**
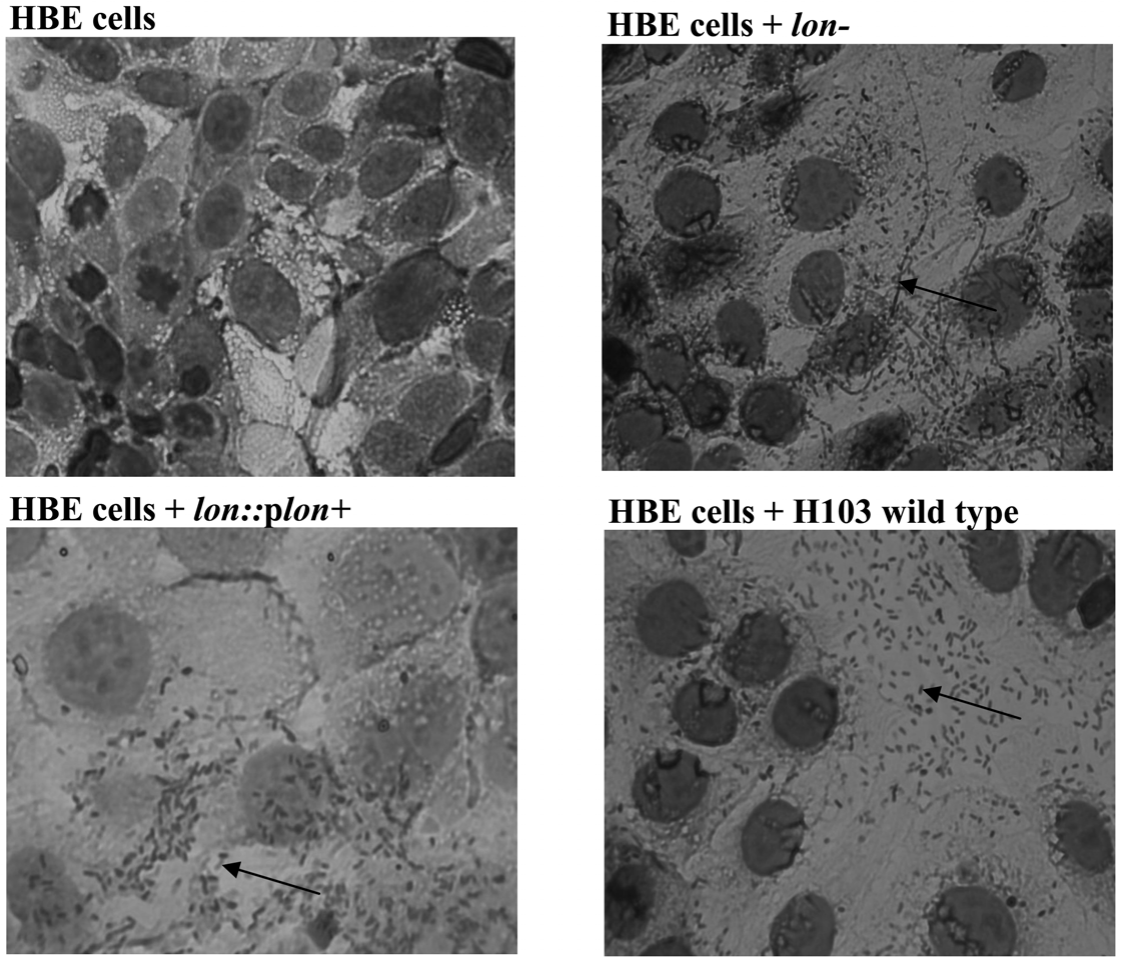
HBE cells infected with *P. aeruginosa* wild type, *lon* mutant (*lon*-) and complemented strain (*lon*::p*lon*+). HBE cells were co-cultured with bacteria cells for 3 hours to allow adherence. After 3 hours, non-adhered bacteria were removed by washing 3 times with PBS. Remaining bacteria and HBE cells were heat fixed and stained with Diff-Quick to allow microscopic analysis. The arrows indicate the bacterial filaments formed by the *lon* mutant and the single cells of the wild type and complemented strain.

Previously we demonstrated that *Pseudomonas lon* mutants had a variety of defects in virulence-associated properties *in vitro* [19]. To determine if we could observe attenuation of the *lon* mutant in cellular models, we examined its ability to avoid phagocytosis by amoebae, to infect and destroy a monolayer of HBE cells and to adhere to HBE cells *in vitro* (Fig. 1). The study of bacterial phagocytosis by amoebae such as *Dictyostelium discoideum*, provides a good cellular model system for studying bacterial virulence [33]. In the amoeba model, a more virulent strain will limit the ability of the amoeba to form a plaque on a bacterial lawn to a greater extent than a less virulent strain. This was assessed as plaque formation (bacterial killing) by varying concentrations of amoebae after 3 to 5 days post-infection for all strains tested. When comparing the wild-type strain PAO1 and the *lon* mutant, the latter displayed a twofold reduction in the number of amoeba cells required to form a plaque, which were 40–60 and 20 for the wild type and the mutant, respectively. Complementation with a wild-type copy of *lon* in pBBR1MCS4 plasmid did not restore the ability of the mutant to withstand phagocytosis to wild-type levels. This was likely due to loss of the plasmid during the incubation. For this reason, the amoeba model was also performed on the PA14 wild-type and *lon* mutant strains in order to confirm the results observed in the PAO1 background. The PA14 *lon* mutant also showed a twofold reduction in the number of amoebae necessary to form a clearing zone, which were 5000 for the wild type compared to 2500 for the mutant strain. Of note, the more virulent PA14 wild type was much more resistant to killing by amoebae than the PAO1 wild type.

In addition to the amoeba model, the cytotoxicity of the PAO1 wild type and the *lon* mutant was also assessed. *Pseudomonas* is known to cause rapid destruction of epithelial cells which is commonly used as a cellular model for cytotoxic potential that frequently correlates with the results observed in animal models. Cytotoxicity was determined by quantifying the amount of cytosolic LDH released by HBE cells into the medium at several time points following infection with *P. aeruginos*a PAO1 wild type or the *lon* mutant. A minor decrease in cytotoxicity was consistently found in the *lon* mutant compared to the wild-type strain at early time-points (data not shown).

Intriguingly, microscopy of bacteria adhered to HBE cells revealed that the *lon* mutant bacteria that had adhered to the HBE cells, tended to form filaments, whereas the wild type and complemented strain adhered to the HBE cells as individual cells (Fig. 1). This might have reduced the overall surface contact of bacteria with HBE cells in the case of the *lon* mutant and, for example, decreased the efficiency of Type III secretion of toxins, which requires direct contact between the host cell and the bacteria, thus leading to the observed reduced damage of epithelial cells. The reduced expression of Type III secretion genes (see below) was also a possible explanation.

### The *lon* Mutation Led to a Major Defect in Growth and Maintenance *in vivo* in a Chronic Lung Infection Model

The importance of the Lon protease in chronic infections was investigated in a rat model of chronic lung infection, chosen due to its relevance to CF, by measuring the relative ability to grow in a mixed culture *in vivo*, assessed as a competitive index.

**Figure 2:**
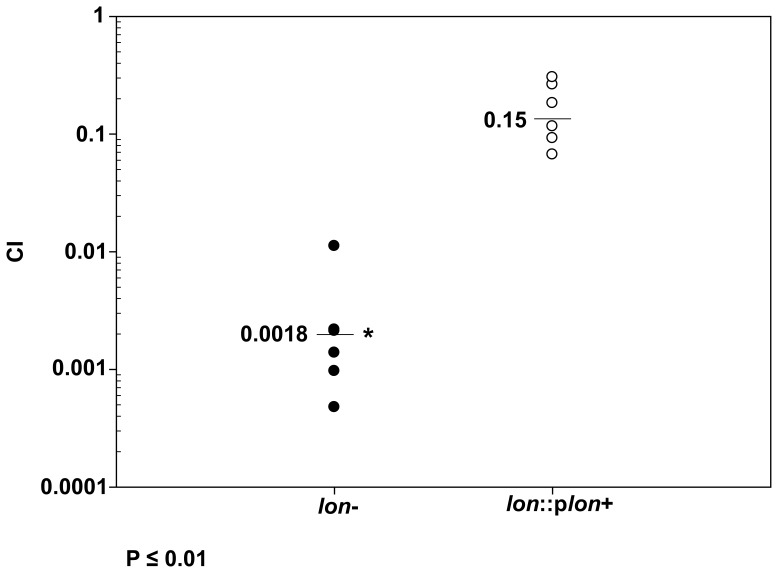
Role of the Lon protease in a rat chronic lung infection model. Equal ratios of the WT PAO1 and *lon* mutant (left) or WT and complemented strain (right) were embedded in agarose beads and delivered to the rat lungs via intubation. After 7 days post-infection, rats were sacrificed and lungs were recovered for CFU determinations. The competitive index was analyzed as described in Methods and is the ratio of the recovered CFU for test strain compared to the WT, corrected for the input numbers of bacteria. Each point represents the competitive index (CI) for a single animal. The *lon* mutant had a major attenuation in growth in this chronic model, which was statistically significant as measured with the Mann Whitney test (p<0.05).

The competitive index was first determined *in vitro* and it was demonstrated that the wild type and the *lon* mutant, as well as the *lon* mutant and the complemented strain, grew at approximately the same rates with *in vitro* competitive indexes of 0.81±0.09 (p = 0.3) and 0.89±0.24 (p = 0.1), respectively, indicating that there was no significant growth difference between the tested strains. In contrast, competitive growth studies in a rat lung model of chronic infection highlighted the importance of Lon in *Pseudomonas* chronic lung infections. The *lon* mutant displayed a >500-fold reduction in maintenance *in vivo* when in competition with the wild type strain (CI of 0.0018, p<0.05) and a 100 fold reduction compared to the complemented strain (p<0.05), when evaluated 7 days post-infection (Fig. 2). This represented a major attenuation in *in vivo* growth of the *lon* mutant. Furthermore, these studies showed that this defect could be largely reversed by complementation with the *lon* gene in *trans;* however, likely due to copy number effects that would change the levels of expression of downstream genes, and/or the potential loss of the plasmid during the experiment, a modest attenuation of competitive growth was observed (CI of 0.15, p = 0.015) for the complemented strain.

**Figure 3:**
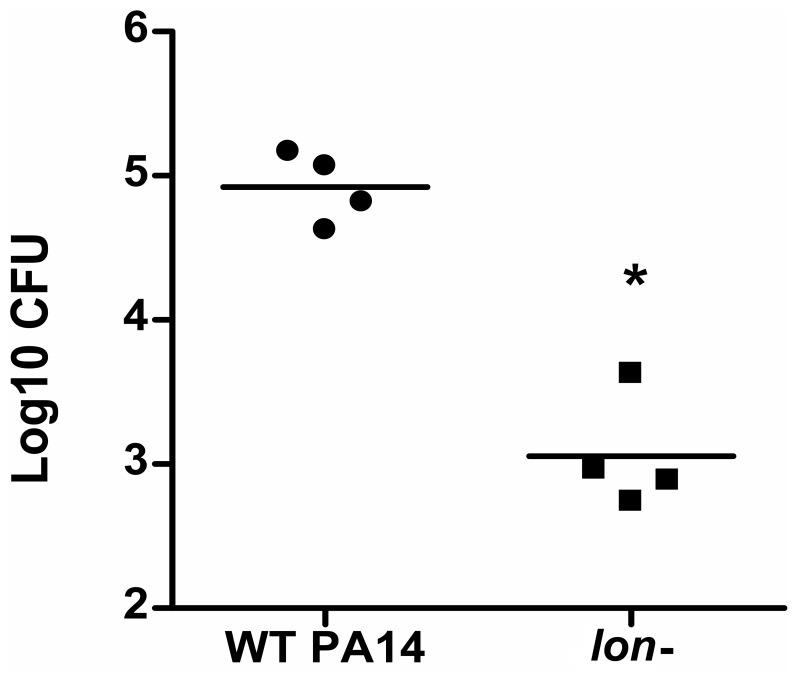
Role of the Lon protease in a mouse acute lung infection model. CD-1 mice were inoculated via intranasal route with 10^6^ CFU PA14 WT or *lon* mutant. Mice were sacrificed 4 hours post-infection and bronchoalveolar lavage fluids were collected for bacterial CFU determinations. Each point represents results from a single mouse and the graph is representative from 3 independent mouse experiments. The *lon* mutant showed a 2 log defect in virulence which was statistically significant (*p<0.05) as assessed using the Mann Whitney test for the experiment shown here and also 3 pooled experiments.

As *Pseudomonas* causes acute nosocomial pneumonia as well as chronic lung infections, we studied the role of the Lon protease in an established murine model of acute lung infection [28], [34]. Mice infected with the *lon* mutant for 4 hours showed only a slight change in their breathing rate, while the animals given the wild-type strain PA14 exhibited severe breathing deficiency and further signs of distress, which are typically associated with an ongoing infection. The bacterial load recovered from the bronchoalveolar lavage 4 hours post-infection revealed that mice infected with the *lon* mutant displayed a 2 logarithm decrease relative to the wild type (p = 0.028, Mann Whitney test) (Fig. 3). This indicted that the Lon protease was important in *Pseudomonas* acute lung infections.

### Attenuation of Type III Secretion in *P. aeruginosa* Deficient in Production of Lon Protease

The early deficiency in cytotoxicity and adhesion provided one possible explanation for the decreased virulence of the *lon* mutant in mouse models. To examine the potential basis for this observation we examined the expression of *P. aeruginosa* virulence factors in the *lon* mutant compared to the wild type using qRT-PCR on bacteria under mid-logarithmic and swarming conditions. These studies indicated that under the tested conditions, the *lon* mutant showed a dysregulation of genes involved in Type III secretion, including the substrate cytotoxin *exoS* (Table 2). Interestingly, the Type III secretion system genes were down-regulated in the *lon* mutant, compared to the wild type, under non-inducing conditions in both mid-logarithmic cultures and cells from swarming colonies. The observed reduced expression of these genes in the mutant was consistent with the observed virulence defect in both the cellular and animal models.

**Table 2:** Gene expression regulation in the *lon* mutant compared to the wild type H103.

PA number	Gene name	Fold change in gene expressionin the *lon* mutant relative towild type at mid-log phase	Fold change in gene expression in the *lon*mutant relative to wild typeunder swarming conditions
**TTSS**			
PA1704-05	*pcrR-G*	−1.7±0.1	−3.6±0.8
PA1708	*popB*	−2.7±1.1	−3.3±0.5
PA1709	*popD*	−4.4±1.1	−4.3±1.4
PA1710	*exsC*	−2.3±0.7	−2.8±0.4
PA1719	*pscF*	−3.8±0.9	−3.2±0.4
PA3841	*exoS*	−2.2±0.5	−2.8±1.2

Gene expression measured with qPCR.

### Role of Lon in Surfing Motility

Our laboratory has demonstrated that in the presence of mucin, a glycopeptide from the mucous layer that covers the airway epithelia, *Pseudomonas* undergoes a unique form of motility, termed surfing motility [35]. Surfing motility is a complex adaptation of *P. aeruginosa* distinct from swarming motility, since surfing motility is flagella but not pilus-dependent and is morphologically and genetically distinct.

**Figure 4:**
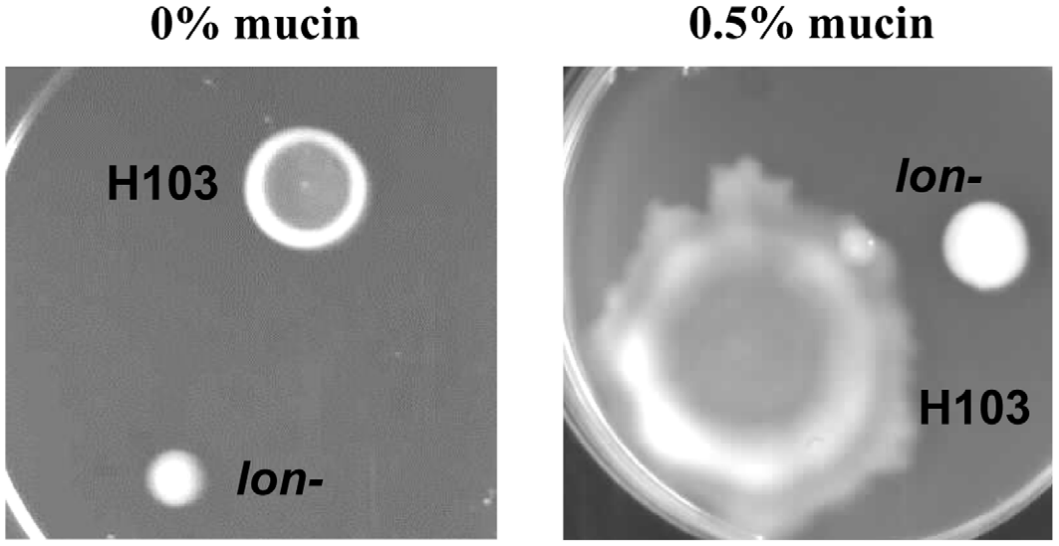
Role of Lon in surfing motility on mucin containing plates. *P. aeruginosa* wild type and *lon* mutant spotted on medium containing mucin allowed for surfing motility (right), the control without mucin is seen on the left. Mucin was added to the medium in an attempt to more closely mimic the lung environment of cystic fibrosis patients.

Due to the observed defects in infection models of the lung, where mucin overlays the epithelia, we examined the effect of *lon* mutation on surfing motility on BM2 swarming media plates containing 0.4% agar and varying concentrations of mucin (0, 0.1 and 0.5%). A significant increase in the motility zone at higher concentrations of mucin for the wild-type PAO1 strain and to a much lesser extent for the *lon* mutant was observed. Nevertheless, the *lon* mutant was highly deficient in both surfing and swarming motility, in contrast to many swarming deficient mutants that demonstrate normal surfing motility [35]. The diameter of the motility zone observed for the PAO1 wild type increased from 11.5 to 46.25 mm when 0.5% mucin (close to the physiological concentration) was added to the medium. However, in the case of the *lon* mutant an increase from 6.25 to only 13.6 mm was observed (Fig. 4).

**Figure 5:**
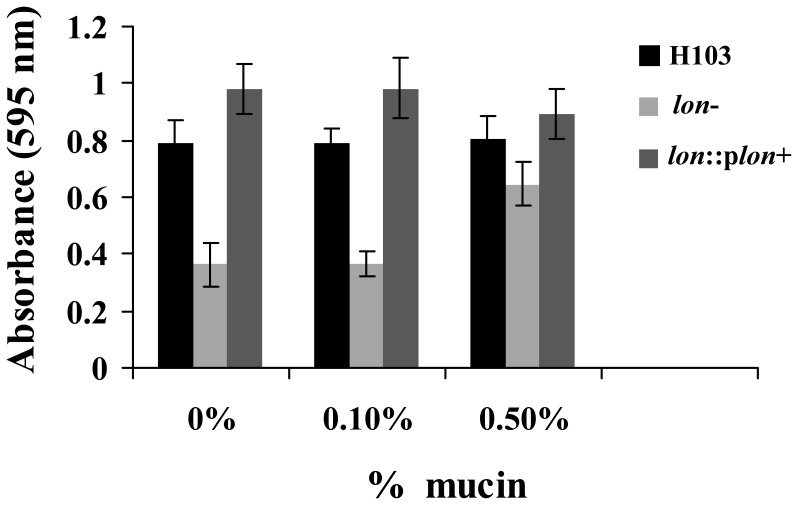
Mature biofilm formation with increasing concentrations of mucin. Cells from the wild-type strain H103, the *lon* mutant (*lon*-) and the complemented strain (*lon*::p*lon*+) were incubated in 96 well microtiter plates containing LB and varying concentrations of mucin (0–0.5%) for 20 hours at 37°C. Biofilm formation was measured by crystal violet staining of the adherent cells. The bars represent the average and standard deviation of 3 independent experiments.

Because of the defect in growth in the chronic rat lung model where bacteria are likely to grow as biofilms, we also tested the effects of mucin on the abiotic biofilm defect of the *lon* mutant. No major differences in rapid attachment were observed for either strain when increasing concentrations of mucin were added to the growth medium. However, mucin at a concentration of 0.5% increased the formation of mature biofilms in the *lon* mutant, but not in the wild type (Fig. 5). On average, the biofilm-deficient *lon* mutant showed an increased ability to form mature biofilms of up to 180% in the presence of 0.5% mucin compared to no mucin, although it was still deficient in biofilm formation compared to the wild-type H103. This difference was statistically significant as determined with Student’s *t* test (p<0.05). The biofilm defect could be complemented by introducing the *lon* gene in *trans*. No differences were observed for either strain when 0.1% mucin was added to the growth media. Mucin also had an impact on antibiotic resistance. Thus, the addition of mucin caused a twofold increase in the ciprofloxacin MIC from 0.1 µg/ml to 0.2 µg/ml and from 0.0125 µg/ml to 0.025 µg/ml for the wild type and the mutant, respectively.

## Discussion

Previous studies had already demonstrated that mutations affecting the *lon* gene lead to defects in motility and biofilm formation *in vitro* [19]. However, the participation of the Lon protease in *P. aeruginosa* virulence *in vivo* had not been established yet. This was accomplished here by using *in vivo* models and investigating the influence of the *P. aeruginosa* Lon protease on interaction with epithelial cells and phagocytic amoebae.

Our results provided insights regarding the importance of the Lon protease of *P. aeruginosa* in virulence. We examined the ability of the *lon* mutant to initiate and maintain pulmonary infections in two *in vivo* models: a short term mouse model of acute lung infection and a longer term rat model of chronic lung infection. The early stages are essential in establishing an infection, and for lung infections this includes the progress of the pathogen through the respiratory tract as well as its adherence to the epithelium, while resisting innate immune clearance mechanisms. Within 4 hours of infection, mice inoculated with the *lon* mutant displayed a significant lower bacterial load in the BAL compared to that for the PA14 wild type, highlighting the importance of the Lon protease during the initial stages of the infection. In addition, results obtained from the chronic lung infection indicated that the Lon protease also played a role in bacterial colonization within the lung. Rats infected with the mutant exhibited a major reduction in the ability to colonize the lungs.

Due to the complex effects of Lon with more than 230 genes dysregulated (Breidenstein and Hancock, unpublished data), we did not examine the specific genes involved in reduced virulence. However, this is quite consistent with the reduction of several *in vitro* virulence-related properties including swarming (and surfing) motility, biofilm formation [19] as well as the suppression of expression of Type III secretion genes, and reduced cytotoxicity against epithelial cells as observed here.

For example, we demonstrated that *lon* mutants were deficient in cytotoxicity towards HBE cells compared to the wild type at early time points (10 hours post-infection). This observed deficiency was consistent with the decreased expression of Type III secretion genes. The Type III secretion system of *P. aeruginosa* is involved in contact-dependent secretion of cytotoxins such as ExoS, and consequent cytotoxicity [36]. Here we observed downregulation of selected genes involved in the different aspects of Type III secretion, including *pscF* (needle complex), *popB* and *popD* (translocation apparatus), *exsC* (regulatory protein) and *exoS* (effector protein cytotoxin), in the *lon* mutant compared to the wild type in mid-logarithmic phase and during swarming conditions. Consistent with these data, the Lon protease, alone or in combination with ClpP, has been shown to affect Type III secretion in *Yersinia* [37]. Since the defect in cytotoxicity for the *P. aeruginosa lon* mutant was observed only at early time points, it seems possible that cumulative secretion of the toxins over time override the modest decrease in expression of this system, or variously at later time points, other secretion and adhesion genes independent of Lon become more influential, leading to an absence of a cytotoxicity defect in the *lon* mutant. In addition to this, a phenotypic defect was observed whereby the *lon* mutant adhered to HBE cells as long filamentous chains, consistent with the role of Lon in cell division.

Furthermore, neutrophils are rapidly mobilized to ensure bacterial killing after pathogen internalization [34]. The Type III secretion system promotes host cell death, inhibits the respiratory burst and phagocytosis by neutrophils [38], [39]. We can therefore speculate that the Lon protease may inhibit phagocytosis through the dysregulation of Type III secretion. However, a study that focused on the plant pathogen *P. syringae* showed the opposite dysregulation pattern of Type III secretion effectors in a *lon* mutant strain [40]. Thus, effector proteins such as Hrp and AvrPto were upregulated in this mutant highlighting that the Lon protease negatively regulates Type III secretion in *P. syringae*. Moreover, while deletion of the *lon* gene led to a decrease in virulence in *P. aeruginosa* and other pathogens, Bretz et al. [40] showed the opposite phenotype in *P. syringae lon* mutants, which is in good agreement with the upregulation of virulence factors. Indeed, tissue necrosis could be observed after only 3 hours of infection with the *lon* mutant, whereas 6 hours were necessary to see a necrotic phenotype for the wild type. In conclusion, the phenotypes observed in *P. aeruginosa* and *Yersinia* stand in contrast with the results obtained in *P. syringae*, indicating that regulation mediated by the Lon protease must occur differently in different pathogens. Nevertheless, all these studies consistently show that Lon exerts an influence in the expression of Type III secretion effectors and appears to play a critical role in the virulence of pathogenic bacteria.

Our observations of decreased virulence of the *lon* mutant in *P. aeruginosa* correlated with observations made in *Salmonella* and *Campylobacter* [14], [15], [16], suggesting that the Lon protease has a common role in determining bacterial virulence. For example, *lon* mutants in *S. enterica* did not cause lethal systemic disease of mice due to their inability to proliferate within the spleen and decreased viability in murine macrophages over a period of 48 hours, consistent with a reduced ability to withstand the bactericidal mechanisms of macrophage killing [16]. Nevertheless, a *S. enterica lon* mutant was able to cause persistent infection in mice, although it did not multiply as efficiently as the wild type [16]. Similarly, the *P. aeruginosa lon* mutant was able to grow in a chronic rat lung model, but had a severe competitive growth disadvantage. In this regard its defective ability to form biofilms would likely influence persistence in a chronic model.

Another goal of this study was to attempt to mimic the CF lung environment by adding mucin to the growth medium. Mucin has already been demonstrated to serve as an energy and nutrient source, is required for attachment of *Pseudomonas* [41] and promotes surfing motility [35]. We wanted to relate our *in vitro* data to the rat model of chronic infection, as high amounts of mucin are a characteristic of a chronic lung infection and we could show that the Lon protease is also important for surfing motility. Apart from promoting surface motility, the addition of 0.5% mucin led to an increase in ciprofloxacin resistance. This finding is particularly significant since ciprofloxacin is currently used for treatment of *P. aeruginosa* infections in CF.

Overall, we demonstrated that the Lon protease is important for full virulence of *P. aeruginosa,* and that the presence of mucin, which is an important component of the CF lung environment, resulted in an increase in ciprofloxacin resistance and surface motility. Particularly interesting was the fact that the Lon protease plays an important role in both types of infections with which *Pseudomonas* has been associated, acute and chronic infections. The dual role of Lon in determining virulence and ciprofloxacin resistance indicate that the Lon protease might be a good therapeutic target to address recalcitrant infections caused by *P. aeruginosa*.
